# Endothelin antagonists for hypertension: has their time finally arrived?

**DOI:** 10.1042/CS20255853

**Published:** 2025-07-03

**Authors:** Ernesto L. Schiffrin

**Affiliations:** Hypertension and Vascular Research Unit, Lady Davis Institute for Medical Research and Department of Medicine, Sir Mortimer B. Davis-Jewish General Hospital, MGill University, Montreal, Quebec, Canada

**Keywords:** bood pressure, endothelins, hypertension, target organ damage, vascular remodeling

## Abstract

There have been few new treatments introduced for hypertension in the last thirty years. The endothelin (ET) system was discovered in 1988, and in the following years, it was demonstrated that it participated in the elevation of blood pressure and vascular remodeling in experimental models of hypertension, particularly those with more severe forms of hypertension. Several selective and dual antagonists of ET_A_ receptors (ET_A_Rs) and ET_B_ receptors (ET_B_Rs) were developed, but none reached marketing for human hypertension. Following a successful trial in resistant hypertension, a novel antihypertensive agent has been approved in Europe and in the U.S.A.: the dual ET_A_R/ET_B_R antagonist aprocitentan, which was recently approved for the treatment of hypertension in combination with other antihypertensive drugs, to lower blood pressure in adult patients who are not adequately controlled on other drugs. Thus, the time has finally arrived for ET antagonists in hypertension.

## Introduction

There have been few novel drugs introduced for the treatment of hypertension in the last thirty years. For the first time in all these years, a totally new antihypertensive agent has now been approved in the U.S.A. and in Europe: the dual endothelin (ET) subtype A receptor (ET_A_R) and ET subtype B receptor (ET_B_R) antagonist (ET_A_RA/ET_B_RA) aprocitentan. It has been approved for the treatment of hypertension in addition to other antihypertensive drugs, to lower BP in adult patients who are not adequately controlled by maximally tolerated doses of first-line antihypertensive agents, including a diuretic. The approval is the result of the development of an ET receptor antagonist (ETRA) that blocks both ET_A_R and ET_B_R and is effective in lowering BP with few adverse side effects [1]. Above all, however, it was the consequence of the positive results of aprocitentan to lower BP and proteinuria in patients of different ethnicities with resistant hypertension in the PRECISION trial [2,3]. Thus, the time has finally arrived for ETRA in hypertension.

ET is a 21-amino acid peptide discovered in 1988 by Yanagisawa et al. [4]. ET-1, -2, and -3 are isopeptides with different functions and tissue distribution. There are also larger 31- and 32-amino acid peptides. ET-1 is the most abundant ET peptide produced primarily by the vascular endothelium and in kidney epithelial cells [5]. ET-3, on the other hand, is a neuropeptide involved during embryogenesis in the migration of cells from the neural crest to the myenteric plexus, meaning that if they do not migrate in the case of an ET-3 gene knockout, congenital megacolon will result (Hirschsprung’s disease). In endothelial and other cells, furin and other enzymes generate 38–39-amino acid peptides, the big ETs, by acting on proendothelins. These are then converted into the 21-amino acid ET-1 by zinc-dependent endoproteases called ET-converting enzymes (ECEs), of which there are two: ECE-1 and ECE-2. ECEs will cleave big ET-1 at the Trp^21^–Val^22^ bond to generate the final product, ET-1. As a result of the existence of four alternative promoters, four differentially spliced isoforms of ECE-1 are encoded by a single gene in endothelial cells. They have different N-terminal amino acids that lead to differential cellular localization. ECE-1a, c, and d are extracellular, whereas ECE-1b is found intracellularly. ECE-1b heterodimerizes with other ECE-1 isoforms and regulates their activity. ECE-2 in smooth muscle cells converts big ET-1 to ET-1 close to ET receptors (ETRs), and accordingly, ET-1 is protected from degradation. An additional pathway comprises matrix metalloproteinase-2, which generates ET-1 by cleaving the Gly^32^–Leu^33^ bond. Mast cell chymase is another enzyme involved in releasing ET-1 by cleaving big ET-1 at the Tyr^31^–Gly^32^ peptide bond. Finally, neutral endopeptidase can also release ET-1. However, the physiological role of these enzymes remains unclear [5].

**Figure 1:**
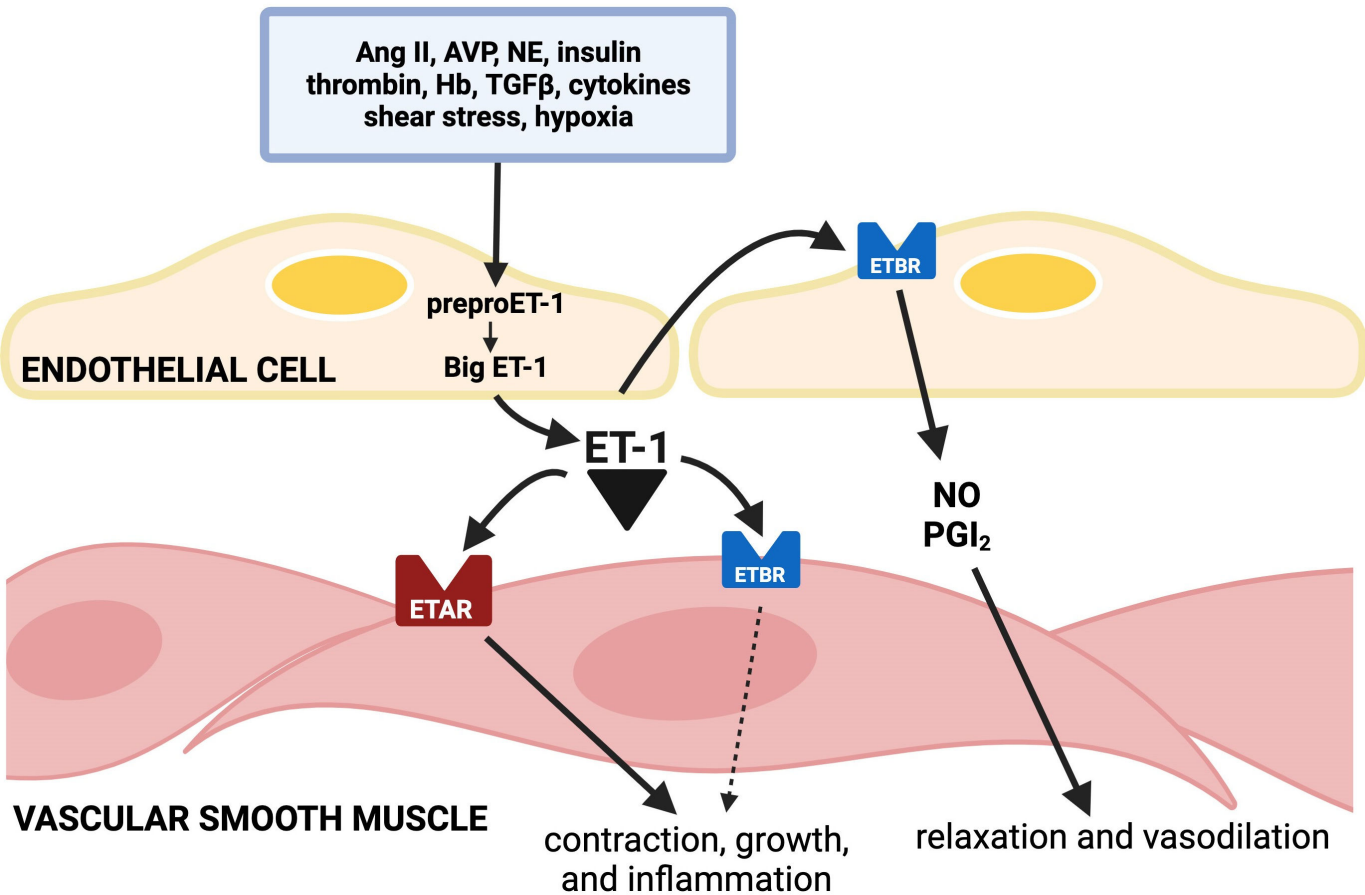
The vascular endothelin system is depicted ET-1 (endothelin-1) produced in endothelial cells from proET-1 acts on vasoconstrictor ET_A_ receptors (ET_A_Rs) and on the underlying vascular smooth muscle in the wall of blood vessels. ET-1 is also secreted abluminally into the bloodstream and acts on endothelial ET_B_R (autocrine effect) to stimulate the production of NO and prostacyclin. Ang II, angiotensin II; AVP, arginine vasopressin; ET_B_R, ET_B_ receptor; Hb, hemoglobin; NE, norepinephrine; PGI_2_, prostacyclin; TGF*β*, transforming growth factor β. Reproduced from reference 5

The secretion of ET-1 by endothelial cells is stimulated by thrombin, glucose, heme, epinephrine, angiotensin II (Ang II), vasopressin, insulin, leptin, and cytokines and growth factor-β1, and by hypoxia, acidosis, or low shear stress (Figure 1). The release of ET-1 is inhibited by increased shear stress and by prostacyclin and nitric oxide (NO) [5,6]. The production of ET-1 has also been demonstrated after T cell activation in monocytes stimulated by immune mediators such as interferon-γ and tumor necrosis factor-α [7].

ET-1 is one of the most potent vasoconstrictors in nature by its action on ETRs, both via ET_A_R and ET_B_R (Figure 1) [5,6]. ET-1 also induces an inflammatory and cell growth response via ET_A_R and ET_B_R on smooth muscle cells in the vascular wall. On endothelial cells, ET-1 acting via ET_B_R stimulates the release of the vasodilators NO and prostacyclin. Heterozygous ET_B_R knockout mice exhibit slight elevations of BP that are exacerbated by a high-salt diet. This has suggested that the physiological action of ET-1 via ET_B_R could be vasodilation, despite the fact that ET-1 is one of the most powerful vasoconstrictors in nature. However, it is likely that the vasoconstrictor action of ET-1 exerted via ET_A_R or ET_B_R on smooth muscle raises BP in susceptible individuals and contributes to hypertension [8].

ET_A_Rs or ET_B_Rs can be detected in many organs beyond blood vessels, including adrenal glands, in which ET-1 stimulates the secretion of aldosterone. In the kidney, ET_B_Rs in renal tubules induce natriuresis [5]. In our inducible model of human ET-1 overexpression in endothelium after 3 months, we found inflammation in the kidney and reduction in renal blood flow associated with early renal injury demonstrated by increased kidney injury molecule-1 (KIM-1)expression in renal cortex tubules [9]. There was associated increased myeloid (CD11b^+^) and myeloid-derived suppressive cell (CD11b^+^Gr-1^+^) renal immune cell infiltration, and a greater frequency of myeloid and renal cells expressing the pro-inflammatory marker CD36. ET_B_Rs in the kidney are mainly found in the principal cells within the inner medullary collecting duct [10]. High-salt diets stimulate ET-1 production within the kidney as in other cells. ET_B_R stimulation results in the production of NO and cGMP that mediate the effects of ET-1 as in other cells, and which will inhibit epithelial Na channel to enhance natriuresis and diuresis. Pro-fibrotic and pro-inflammatory actions appear to be exclusively mediated by ET_A_R activation, as demonstrated by blockade using the ET_A_RA atrasentan in our inducible human ET-1-producing mouse, as described above [9].

ET in the brain is produced in rostral areas including the subfornical organ (SFO) [11]. The SFO exerts effects on the hypothalamus and the brainstem, whereby the ET system stimulates cardiovascular regulatory centers. Thus, ET via ET_A_Rs and ET_B_Rs in the ventrolateral medulla, the area postrema, and nucleus tractus solitarius stimulates sympathetic outflow and regulates heart rate, vascular tone, kidney blood flow, renin secretion, water, and sodium handling, as well as catecholamine release by the adrenal medulla, all of which may have an impact on BP regulation. However, in the central and peripheral nervous systems, ET_B_Rs are the predominant ETRs [5,11]. In hypertensive patients, infusion of an ET_A_RA reduced sympathetic nerve activity in both normotensive and hypertensive individuals, although effects were greater in the latter. In resistant hypertensive subjects treated with renal denervation, plasma ET-1 levels in both renal arteries were reduced [12]. It has also been suggested that ET_B_Rs on adrenergic nerves contribute to baroreflex dysfunction [11].

### Mechanism of action of endothelins

ET_A_Rs or ET_B_Rs are G-protein-coupled receptors that act via stimulation of phospholipase C, inositol trisphosphate generation, and calcium release, activating calmodulin, diacylglycerol production, and protein kinase C stimulation [5,6]. As with other similar G-coupled receptors, ETRs stimulate reduced nicotinamide adenine dinucleotide phosphate oxidase, xanthine oxidase, mitochondria, and uncoupled NO synthase to generate reactive oxygen species and growth factor receptor transactivation. Mitogen-activated kinase (MAPK) stimulation is induced, the ras-raf-MAPK cascade and non-receptor tyrosine kinases are activated, and together with calcium release, vasoconstriction and growth occur. ET_A_R and ET_B_R on vascular smooth muscle may stimulate cell growth, whereas ET_B_R on endothelial cells exerts countervailing actions by stimulating the production of the vasodilators NO and prostacyclin, and on other cells by inducing apoptosis via NFκB activation.

#### Role of endothelins in hypertension

The ET system plays a pathophysiological role in numerous conditions, including primary pulmonary hypertension (the only approved indication of ETRA in humans until the recent approval of aprocitentan for hypertension), atherosclerosis, coronary artery disease, cardiac hypertrophy and heart failure, subarachnoid hemorrhage and cerebral vasospasm, diabetes, pulmonary fibrosis, scleroderma, diabetic and non-diabetic renal disease, hepatorenal syndrome, glaucoma, prostate cancer and its metastasis, and other cancers. However, this review will only concentrate on the role of ET in hypertension.

ET has been implicated in the pathophysiology of salt-dependent hypertensive rodent models such as DOCA-salt hypertension [13] and salt-loaded stroke-prone spontaneously hypertensive rats (SHR) [14], in which enhanced production of ET-1 is induced and leads to BP-independent hypertrophic remodeling of small arteries [15,16]. ET-1 expression in the endothelium of blood vessels was enhanced in many organs, including the heart, associated with an inflammatory response and fibrosis [17]. We have expressed human prepro-ET-1 in mice restricted to the endothelium using the endothelium-specific promoter Tie-2 and reported that there was a minor elevation of BP in these mice associated with small artery hypertrophic remodeling, vascular inflammation, and endothelial dysfunction [18,19], which demonstrates that ET-1 can indeed induce growth effects on blood vessels independently of BP elevation. These mice have enhanced vascular lipid biosynthetic enzyme gene expression and accelerated atherosclerosis, and slightly higher BP elevation when crossed with apoE knockout mice and fed a high-fat diet [20]. They also develop abdominal aortic aneurysms associated with accelerated atherosclerosis, suggesting a role of ET-1 in aneurysm progression [21]. When diabetes is induced with streptozotocin, these mice with endothelial ET-1 overexpression exhibit enhanced oxidative stress mediated by NOX-1 and inflammation, leading to exaggerated progression of atherosclerosis [22]. Inducible endothelium-restricted human ET-1 overexpressing mice (i.e. ET-1) present elevation of BP at three weeks [23], which is sustained for at least three months, associated with vascular and renal injury and inflammation [9], all reduced by treatment with the ET_A_R blocker atrasentan.

Interestingly, early in the investigation of the mechanisms of action of ET-1, it was demonstrated that the ET system had a close relationship with the renin-angiotensin-aldosterone system. Using cultured vascular smooth muscle cells, Rajagopalan et al. [24] showed that Ang II stimulated the expression of ET-1. Also, Ang II infusion in rats increased ET-1 immunostaining in vascular smooth muscle ET-1 and its plasma levels. The selective ET_A_R PD 155080 blocked the hypertensive response to Ang II. Hyperresponsiveness to several vasoconstrictors except ET-1 was enhanced by Ang II-induced hypertension and blocked by the ET_A_RA. The authors concluded that vascular effects of Ang II are mediated at least in part by ET-1.

Aldosterone infusion increased the expression of ET-1 in endothelial cells [25]. With greater recognition of the role of hyperaldosteronism in human hypertension [26] and particularly in resistant hypertension [27], this may contribute to explaining a role of ET in primary hypertension in humans, even though plasma ET levels are not elevated in primary hypertension [28]. Also, other contributory factors could be the rapid clearance of ET from the circulation together with the fact that the secretion of ET-1 occurs from endothelial cells toward underlying smooth muscle with little spillover into the circulation [29]. Nevertheless, elevated levels of ET-1 in the circulation have been reported in African Americans [30] who have a low-renin volume-expanded form of hypertension, which may be comparable to the DOCA/salt model. Among salt-depleted salt-sensitive hypertensives, some subjects who exhibited blunted responses of renin to salt depletion had increased catecholamine-stimulated ET-1 concentrations in plasma, suggesting that they could respond to ETRA with BP lowering [31]. Furthermore, Cardillo et al. have reported a role of ET-1 in the increased vascular tone of patients with essential hypertension [32]. In difficult-to-control hypertension in humans, we have indeed demonstrated that the expression of prepro-ET-1 is enhanced in the endothelium of small arteries [33].

An additional mechanism that has been reported to enhance ET-1 action, which could raise BP through effects on sodium excretion, results from the loss of ET_B_R as demonstrated by Johnston et al. [34]. Since ET-1 regulates sodium balance by promoting natriuresis through ET_B_R in response to increased salt in the diet, these authors tested the hypothesis that ET_B_R activation contributes to the diurnal control of sodium excretion, and that sex differences contribute to this control as well. Using male and female ET_B_R-deficient (ET_B_R-def) rats, they demonstrated impairment of natriuretic responses to a salt load during inactivity in male but not female ET_B_R-def rats. Treatment with ET_A_RA ABT-627 improved the natriuretic response in both sexes. The authors concluded that diurnal excretion of an acute salt load requires ET-1 and ET_B_R, that it is more evident in male than female rats, and that it is blocked by ET_A_RA. The authors suggest that there is a direct link between circadian rhythms and the regulation of sodium excretion via ET_B_R, different in males and females, which could contribute to BP regulation.

### Effect of ETRA on models of experimental hypertension

The dual ET_A_RA/ET_B_RA bosentan lowered BP and reduced vascular remodeling, in the DOCA/salt-treated rat [15] and in salt-treated stroke-prone SHR [14]. Small arteries from the coronary, renal, and mesenteric circulations showed a smaller media width and cross-sectional area of the media in rats treated with bosentan than in untreated rats. The kidneys showed the presence of fibrinoid necrosis in a high percentage of afferent arterioles and glomeruli of DOCA-treated SHR. Some kidneys of treated rats exhibited less severe vascular hypertrophy and lesser extent of vascular or glomerular fibrinoid necrosis. These results suggested a role for ET-1 in BP elevation and the severe vascular hypertrophy of small arteries of the coronary, renal, and mesenteric vasculature, but not of the heart or larger conduit vessels in the malignant hypertension that SHR develop after treatment with DOCA and salt. 35Thus, in general, it was the more severe forms of experimental hypertension that exhibited enhanced ET-1 expression and responded to ETRA with lowering of BP. Together with enhanced vascular tone in hypertensive humans [32] and exaggerated expression of ET-1 in the endothelium of small arteries in humans with uncontrolled hypertension [33], this suggested that hypertensive patients with difficult-to-control hypertension would be prime candidates for treatment with ETRA.

### Effect of ETRA in humans

The first study using ETRA in hypertensive humans was carried out in 293 patients with mild-to-moderate essential hypertension with bosentan, the first dual ET_A_RA/ET_B_RA available. BP was lowered by 5.7 mmHg by 500 mg once daily or 1000 mg twice daily of bosentan, which was similar to the effect of 20 mg of enalapril once daily, over four weeks of treatment [35]. However, although bosentan lowered BP, it had adverse side effects, including liver enzyme elevation, that precluded its use in a chronic disease such as primary hypertension, which may last for decades. It was reserved for primary pulmonary hypertension, a rapidly fatal disease, and the same applied to other ETRs that were developed later. When a clinical trial was carried out with the ET_A_RA darusentan in resistant hypertension, BP in the clinic was not statistically significantly lowered more by the ETRA than by the placebo [36]. Twenty-four-hour ambulatory BP was nevertheless significantly lowered relative to placebo and similarly to the effect of guanfacine, which was used as a comparator in this trial [37]. Despite these positive results, the development of darusentan was abandoned by the pharmaceutical company.

### The PRECISION trial

It is only with aprocitentan, a dual ET_A_RA/ET_B_RA, which was devoid of major adverse side effects [1], that an ETRA has finally arrived in the clinic. Aprocitentan was tested on resistant hypertensive patients in the PRECISION trial [38]. This Phase 3 trial was carried out in Europe, North America, Asia, and Australia. It was a multicenter, randomized, parallel-group study [3]. Patients with a sitting systolic BP of 140 mmHg or higher, who were receiving maximal tolerated doses of three first-line antihypertensive agents that included a diuretic, were recruited. The study had three parts. The first was a double-blind, randomized, placebo-controlled four-week part, in which patients received aprocitentan 12.5 mg, aprocitentan 25 mg, or placebo in a 1:1:1 ratio. The second part was 32-week long and consisted of a single-blind period (for the patients) in which they received aprocitentan 25 mg daily. The third part lasted for 12 weeks and was a double-blind, randomized, placebo-controlled withdrawal period, in which the patients were randomized again to aprocitentan 25 mg or placebo in a 1:1 ratio. The primary endpoint was the change in unattended automated office systolic BP from baseline to week 4. The major secondary endpoint was the change in systolic BP from withdrawal baseline to week 40. An additional secondary endpoint included changes in ambulatory BP. A total of 1965 individuals were screened and 730 were selected [2]. Of these, 96% completed part 1 of the study. Of those who completed part 1, 87% completed part 2. Out of the ones who completed part 2, 94% completed the third part of the study. The least-square mean difference versus placebo in office systolic BP at four weeks for aprocitentan 12.5 mg and 25 mg was –3.8 mmHg (*P*=0.0042) and –3.7 mmHg (*P*=0.0046), respectively. The respective difference for 24-h ambulatory systolic BP was –4.2 mmHg and –5.9 mmHg for aprocitentan 12.5 mg and 25 mg, respectively, relative to placebo. After the withdrawal of aprocitentan, office systolic BP significantly increased with placebo versus aprocitentan by 5.8 mmHg (*P*<0.0001). Adverse events were mild-to-moderate edema or fluid retention in less than 18% of patients receiving aprocitentan 12. 5mg, 25 mg, and placebo, during the four-week double-blind period, and resulted in discontinuing aprocitentan in seven patients. There were 11 deaths during the study, which were not considered to be related to the treatment. A recent publication has demonstrated that in the PRECISION trial, African Americans benefited equally to Caucasians, [3] although placebo effects in this population were much larger than in the whole cohort for reasons that could not be established. Based on the PRECISION trial, the FDA and the European Medicines Agency have approved aprocitentan at a dose of 12.5 mg once daily for treatment of adults for control of uncontrolled hypertension on top of maximally tolerated doses of first-line agents, including a diuretic.

Recently, the idea of adding diuretics or sodium-glucose cotransporter-2 inhibitors such as dapagliflozin as a single pill combination could allow some of the more frequent adverse side effects such as edema or fluid retention induced by treatment with ETRs such as aprocitentan to be prevented.

### Will endothelin system molecular genetics help indicate who are the hypertensive patients who will respond best to ETRA?

The association of polymorphisms in the coding region of the prepro-ET-1 gene (*EDN1 K198N*) has been reported with increases in vascular reactivity and BP in obese individuals [39]. Also, a polymorphism in the 5′-regulatory region of the ECE-1b gene *ECE1 C-388A* is associated with increases in the expression of ECE-1b and the generation of ET-1 [40]. The A allele was shown to affect daytime and night-time BP in untreated hypertensive German women and in women in the French epidemiological study Étude du Vieillissement Artériel [41]. In the latter study, however, the *EDN1 K198N* polymorphism was not associated with BP in men or women. Nevertheless, an interaction was reported with *ECE1 C-338A* that influenced systolic and mean BP levels in women. Interestingly, the *EDN1 K198N* polymorphism is associated with the potentiation of vasoconstrictor effects at low concentrations of ET-1.

A genetic variant associated with five vascular diseases has been reported to be a distal regulator of *EDN1* expression [42]. The variant responsible for increased risk of coronary artery disease, as well as lower risk of migraine, carotid dissection, fibromuscular dysplasia, and hypertension, is a common single nucleotide polymorphism in the third intron of the *PHACTR1* gene, *rs9349379*. However, ET-1 plays a role in experimental and human hypertension (see above), which could depend on the genetic background of those who develop hypertension in response to ET-1. Thus, increased ET-1 vasoconstrictor action mediated by ET_A_R and ET_B_R on vascular smooth muscle could counter the vasodilator action of endothelial ET_B_R and result in elevated BP [7].

## Conclusion

ET-1 is a potent vasoconstrictor that promotes cardiac, vascular, and renal inflammation, hypertrophy, and fibrosis. ETRA could prevent some of the complications of hypertension, atherosclerosis, and diabetes, and it is possible that they could achieve BP-independent cardiovascular protection. However, because of side effects, their potential usefulness in hypertension, heart failure, atherosclerosis, CKD, diabetes, and other diseases has not been exploited. Until recently, the only approved indication of ETR blockade was primary pulmonary hypertension. However, with the recent results with aprocitentan, a dual ET_A_RA/ET_B_RA in resistant hypertension in the PRECISION trial, the time for ETRA in hypertension has finally arrived. The identification of genetic modulators of susceptibility to ETRA provides an opportunity for pharmacogenetic determination of which patients should use these agents to treat hypertension more successfully.

## Abbreviations

Ang II: angiotensin II
ECEs: endothelin-converting enzymes
ET: endothelin
ET_A_R: ET_A_ receptor
ET_B_R: ET_B_ receptor
ETR: endothelin receptor
ETRA: ET receptor antagonist
MAPK: mitogen-activated kinase
NO: nitric oxide
SFO: subfornical organ
SHR: spontaneously hypertensive rats
